# Hospital and Institutional News

**Published:** 1916-08-05

**Authors:** 


### HOSPITAL AND INSTITUTIONAL NEWS.

### LORD KNUTSFORD'S RETURN TO HEALTH.

That Lord Knutsford has returned to work on recovery from his severe accident of some months ago is amply attested by his new appeal on behalf of the London Hospital, an appeal which has received the unusual endorsement of a leading article in the *Times*. In spite of the success of the usual quinquennial appeal not so very long ago, the finances of the London Hospital have reached such a crisis that, failing a generous public response to this appeal, it is suggested that either the quantity or the quality of the work done must suffer. During Lord Knutsford's illness a committee of business men, aided by experts, went through the expenditure of all the departments of the hospital; their report indicates that economy and reduction of expenses cannot be carried any further, save as just indicated. By a curious coincidence, the *Times* endorsed a similar appeal on behalf of the London Hospital exactly a hundred years before the entry of Britain into the present war—namely, on August 4, 1814.

### WOMEN MEDICAL STUDENTS AND EDINBURGH UNIVERSITY.

The Edinburgh University Court, which has under consideration the admission of women medical students to study and instruction in the University in accordance with the recommendations of the Committee of the Court, has received a letter from Mrs. Charles Watson, the first woman medical graduate, and Miss Mair, chairman of the Edinburgh Hospital and Hospice for Women and Children, containing an offer on behalf of the women medical graduates, students, and their friends of a contribution of £4,000 towards the necessary outlay for providing the facilities of study. There are three conditions attached, which amount to a demand for complete equality with the male students and for an immediate decision. The Court has virtually accepted the offer, by declaring in its resolution of thanks that the scheme is thereby greatly facilitated; but the final decision is postponed until the end of August, when a further report by the committee will be presented.

### A HOME FOR DISABLED OFFICERS.

At a luncheon held at the Ritz Hotel to enlist national support for the Lord Kitchener Memorial Fund, Lord Derby announced that the executive committee had been offered, and had accepted, one of the finest houses in Regent's Park as a home for disabled officers. The building is to be fitted with all the most modern appliances, and Sir Frederick Treves will supervise it, while Sir David Ferrier will be consulting physician. At the same meeting it was declared that if the fund is to be really effective, far larger sums than those hitherto subscribed—liberal though they are—will be required. It looks as if Regent's Park, already the locality of the celebrated St. Dunstan's institution for blinded soldiers, was to become a sort of headquarters for dealing with the wreckage of modern warfare. It was laid out, of course, just about a hundred years ago, when the Napoleonic campaigns were approaching their conclusion; so there is something appropriate in the usage to which it is now being devoted, a century later.

### A RECONSTRUCTED MEDICAL SERVICE.

The Vice-President and Chairman of Council of the National Medical Union, Mr. V. T. Greenyer, F.R.C.S., has put forward a scheme for what is in essentials a State Medical Service for the genuinely necessitous classes of the community, and for them alone. The outlines of this scheme have been embodied in a pamphlet which Mr. Greenyer is publishing privately: from which it may be inferred that the scheme does not bind the National Medical Union in any way. The pamphlet is too long for detailed analysis; but the author starts from the failure of the medical benefits of the Insurance Act, a failure which is pretty generally admitted. Although he does not express it so bluntly, he tacitly recognises that proper economy in a Medical Service is incompatible with free choice of doctor, because the latter means competition among doctors for patients, who will go to the man that is most liberal of potions and certificates. His plan is to induce young qualified men to enter the State service for three to five years after qualification, at a wage of five pounds a week, before embarking on individualistic practice. As long as the medical profession is recruited in its present manner, we doubt if this scheme is feasible, though it is certainly interesting and ingenious. And what are hospitals going to do for residents under such a scheme?

### RESIGNATION OF DR. ARMSTRONG-JONES.

The London Mental Hospitals Committee have received with great regret the resignation of Dr. Robert Armstrong-Jones of his appointment of medical superintendent of Claybury Mental Hospital, to take effect in September next. The committee report that he has rendered more than twenty-eight years of honourable service, first as assistant medical officer at Colney Hatch, and latterly as medical superintendent of Claybury Mental Hospital since the opening of that institution some twenty-three years ago. In accepting Dr. Armstrong-Jones' resignation, the committee have placed on record their sincere regret at the ill-health which has rendered his resignation necessary, and their high appreciation of the great and useful work which he has done at the hospital, and of the use which he has made of his high professional attainments for the benefit of the patients, whose interests, they recognise, have always been his first care. Nor is recognition of this eminent alienist's services confined to those who come into direct touch with him in the capacity of employers. Amongst his colleagues, and amongst the profession at large, his reputation stands deservedly high. His contributions to medical literature have been conspicuously successful in really explaining the intricacies of mental medicine; and he has thus benefited far larger numbers of patients than have actually been under his own personal care.

### WITTENBERG AGAIN.

The infamies of Wittenberg and of the arch-malefactor Aschenbach as narrated by the British officers who survived the typhus epidemic there, are supplemented by new particulars given through the *Bureau Documentaire Belge* by Dr. Destrée and Dr. Bolland, two Belgian doctors who were detained by the Germans, in their usual system of flat violation of the Geneva Convention. Dr. Destrée's testimony deals with the period before the arrival of the gallant Fry and his colleagues, and bears out to the full the accounts given by the British doctors. Segregation of infected and non-infected men was repeatedly refused, as were also overalls, bandages, soap; beds, linen, bedding, light, all were lacking. At least five Russian and Belgian doctors caught the disease, though fortunately the mortality was less than amongst their British colleagues. Maltreatment of every description, including serious assaults, which went unpunished, suppression of correspondence, and even of medicines paid for by the prisoners themselves, were rife in this horrible inferno. Abominable barbarities were frequent and unchecked. Verily, the account against the Germans is mounting up: there must be and shall be a settlement in full some day.

### AN ENGLISH MONASTERY AS A HOSPITAL.

The military authorities having asked that the entire complement of beds at Staincliffe Institution Infirmary should be placed at their disposal, a joint committee of the local authorities in the Dewsbury Poor-Law Union have had to seek accommodation elsewhere. They propose that over 100 permanently bedridden patients should be accommodated in the old hospital attached to the institution; another hundred are to be housed in a temporary building to be erected in the grounds. The last proposal is that a hundred beds should be taken in the House of Resurrection, Mirfield, the council of which, subject to ordinary business conditions, have offered the house to the joint committee for the duration of the war. The Community of the Resurrection at Mirfield, whose members, like the Jesuits, for instance, move about in the world, is not only one of the few Anglican Orders, but one of the few monastic buildings in this country to be used as a hospital. The infirmary patients will probably appreciate the pleasantness of the place.

### THE PROBLEM OF BEDS AT MANCHESTER ROYAL INFIRMARY.

As a result of an urgent request from the War Office, which was made early in July, the Manchester Royal Infirmary has placed 150 additional beds at the disposal of the military authorities. With this addition included there are now 422 beds for wounded soldiers in the infirmary, of which 386 were occupied at the end of July. The Board also announce that civilians have not suffered as a result of this increased accommodation for the wounded, and base this assertion on the fact that the waiting-list of civil patients is much lower now than it was at the beginning of the year. As a waiting-list is subject to fluctuations, as the decline mentioned shows, we hope that the board is not content with this piece of good fortune, but is taking active measures to secure the hospital treatment of civilian cases when the waiting-list lengthens again, as it surely will. Even as things are, not civilians, but the wounded, are reaping the advantage of a smaller waiting-list; while the aim should be, not to be thus content with a light average of waiting cases, but to do away with them altogether. The increased need for providing for the wounded makes the treatment of civil cases again a matter to be pressed.

### MR. E. W. MORRIS ON THE EFFECT OF THE INSURANCE ACT.

Special interest was given to the resumed sittings of the Commission of Investigation appointed by the Faculty of Insurance to inquire into the position of National Health Insurance through the evidence given by Mr. E. W. Morris, secretary of the London Hospital, on the effects of the Act. He said that the fear prevailing when the Act was introduced, that the income of the hospitals would fall, had not been realised. But the only approved societies which subscribed were those which had done so before the Act was passed. He thought the societies ought to recognise the benefits they gained from hospital treatment. The witness did not know of any hospital which had been consulted by the Insurance Commissioners. The effect of the Act had been to reduce trivial cases by about half in five years, which enabled the hospital to concentrate on research and preventive work. Though out-patients had decreased from 90,000 to 77,000 at the London Hospital, the number of insured persons among them was practically the same, and the attendances, on the average, larger. While trivial cases were refused so that they might be treated by panel doctors, he thought maternity cases ought to go to the hospital. Fees for confinements in the East End were prohibitive, because the doctors did not want these cases. As to maternity benefit there were nasty rumours (which the Chairman of the Commission stated that he had heard) that landlords would let expectant mothers fall into arrears of rent and then seize the maternity benefit. For some reason Jewish women much preferred to go to hospital. The party papers have taken this evidence as vindicating the success of the Insurance Act. But the claim that the financial fears of hospital men have not been fulfilled is not correct, seeing that it is the hospitals and not the Act which have provided institutional treatment, the cost of which has not yet been repaid to the hospitals by the Government, panel practice is a very doubtful boon, and real benefits from the Acts rather far to seek.

### AN ABORTIVE SCHEME.

The war has provided the latest practical difficulty in the way of the doubtful scheme for a London Jewish hospital. Although, according to the first annual meeting, a start has been made by the expenditure of £6,000 on a building, occupation is confined to the lower floor, which, though not yet available, it is hoped to open as an out-patient department in the autumn. Thus the scheme for a hospital of fifty beds, and the hope of extending this number to 110, are still a long way from realisation. Its claim to a large measure of working-class support was exaggerated, and the scheme never had real life behind it. The alleged unsuitability of hospital treatment for Jews in our great hospitals, where special wards and kitchens for them have long been provided, was as unwise as it was uncalled for. We record what has been done as the fact warrants, but the net result is that the scheme for all practical purposes was and is abortive.

### THE "MODERN HOSPITAL."

The July number is the first of a new volume of our American contemporary, the *Modern Hospital*, the progress of which we have watched with interest since the publication of its first number in September 1913. The journal may be described as a handsome publication, well printed, and on excellent paper; many of the articles are by men and women, medical and lay, who understand what they are writing about, and the illustrations, not excluding those in the advertisement pages, are generally good and helpful. We have from the outset been surprised to note that for practical purposes the plans and illustrations in the *Modern Hospital* are of slight if any value. This arises from the absence of a scale which a technical journal should supply with every plan. In the number before us there is an article by Mr. Warren C. Hill on the new Robinson memorial of the Massachusetts Homœopathic Hospital. Not a measurement can we find in either text, illustrations, or plans. We have held for thirty years, and acted upon the theory, that plans should be drawn to an identical scale and have the scale plainly indicated, otherwise they are nearly useless. We notice that the new hospital described has (like other American hospitals) private-room accommodation, each room sharing with another a bathroom in which there is a pedestal water-closet; this arrangement is obviously open to objection, and we hardly expected to find it in an up-to-date institution. To return to the Journal, upon which we congratulate the editor and publishers, may we suggest that it would be more convenient to number the advertisement pages in Roman letters? At present the reader is apt to get confused when making use of the index.

### HOSPITAL SUPERINTENDENT GETS £3,000 DAMAGES.

Fortunately, in this country, institutions and their officers are not often to be found in Courts of Law, either as defendants or plaintiffs; moreover, libel actions against newspapers are not frequent, because our Press has generally a salutary habit of verifying its information. That these conditions are not world-wide is shown by the action for defamation by Dr. H. B. Ellerton, the medical superintendent of the Goodna Hospital for the Insane, against the *Brisbane Daily Mail*, the hearing of which occupied thirty sessions of the Court. The plaintiff was in the witness-box over six days; when he was appointed in 1909 he submitted a report to the Home Secretary recommending certain improvements as to staff and accommodation, but he denied categorically the allegations as to the later condition of the institution and its inmates when he had become responsible for good order. The chief allegations by the newspaper were that the patients' food was insufficient and uneatable, that they were allowed to suffer exposure through many broken windows, that the attendants were undisciplined, that the heating apparatus was deficient, that the patients were verminous and shockingly neglected, and that these conditions were largely due to the inefficiency of the medical superintendent. Evidence was given on behalf of the defence, which stuck to the plea of justification to the last, by a medical gentleman who had had no experience of psychiatry, but who had been a *locum tenens* at the hospital for three months in 1914. Another witness, who wrote the articles, considered that he was justified in making sweeping statements after a partial inspection of two wards out of eighteen. These legal proceedings followed upon an inquiry by a Royal Commission, the appointment of which was forced by the publication of the articles. Its unfortunate venture cost the newspaper £3,325 and the expenses of the trial.

### THE SUPPLY OF GREENS TO V.A.D. HOSPITALS.

Gifts in money and kind have passed so extensively into the treasury and larder of the V.A.D. hospitals that the latest suggestion for the organisation of a free supply of green vegetables to them is only the latest proposal of a long list. The method proposed is for the farmers to plant a tract of land with greens for the sole use of the nearest V.A.D. hospital, and it is further suggested that the necessary planting and hoeing could be done early or late in the day by the employees on each farm. The National Farmers' Union has its branch in each county, and, as the suggestion emanates from one of them, it seems invidious to point out the difficulties in the way. Nevertheless it can hardly be questioned that farmers are extremely short-handed at the present time, and the difficulty is rather to find sufficient labour to do the necessary work to keep the land in cultivation. Undoubtedly, if the farmers respond to the suggestion, the 30,000 members of the National Union could do a great deal; but while the difficulties are obvious, it would be interesting to know to what extent V.A.D. hospitals have really been as short of green vegetables as is implied by this novel suggestion. Meanwhile, if the farmers granted the land, would it not be possible and practical to organise the planting and hoeing, apart from the depleted ranks of farm labourers, by voluntary help?

### POOR-LAW CENTRALISATION AND THE IRISH PRIESTS.

At the close of their annual retreat at St. Kieran's College, Kilkenny, the priests of the diocese of Ossory have passed a resolution protesting against the proposal of the Local Government Board of Ireland to close the workhouse hospitals of the county and transfer all patients to a new central hospital at Kilkenny. The protest is based on the customary objection to all such reforms—namely, that the patients would suffer by removal from their home surroundings, and from the journey to "a central hospital among strangers." The priests describe the present workhouse hospitals as "the one solitary beneficent provision of the Poor Law in our county," and therefore urge that they should be left undisturbed. If the Irish workhouse hospitals at all resemble the English ones in the non-separated infirmaries the "beneficent provision" which they make for the sick could be very greatly improved. Priests are generally as well intentioned as they are non-conversant with affairs, and if the remedy of amalgamating infirm wards attached to the workhouses into a central institution is more and more forcing itself upon Poor-Law medical officers as the right remedy here, Ireland will hardly be able to escape the same conclusion. The other arguments concerning the patients are disproved by experience.

### ANOTHER "MEDICAL INDEX."

The announcement is made that we are to have another medical index. This time it is styled "A Quarterly Cumulative Index to Current Medical Literature," which is described by the American Medical Association, which has just announced the issue, as a "practical index to original articles in the better and more accessible medical journals—domestic and foreign." It is said that 110 journals have been selected—of these eighty-seven are domestic, and fifteen are published in Great Britain, two in Canada, and one each in Australia, India, China, and Japan. We understand that it is to be issued four times a year, and that *each* new number includes all references in the earlier issues of the current year, combined in one alphabet and brought up to the date of the new part. The fourth and last number for the year is to be issued in cloth covers for permanent reference. Thus, if the latest index is successful, we shall have three valuable aids of reference to current medical literature from American sources—viz., the "Surgeon-General's Index Catalogue," of which one volume is issued annually, the "Index Medicus"—issued monthly and with a valuable yearly index in two alphabets, Authors and *subjects*—and the one just announced. In this connection it is also announced that the customary half-yearly index of authors and subjects issued with the journal of the American Medical Association is to be discontinued.

### MEMORIAL TO A SECRETARY KILLED IN ACTION.

Various interesting staff changes have occurred at the North Devon Infirmary, Barnstaple, saddest and chief of which during the past year has been the death of Captain R. P. Dunn Pattison, formerly honorary secretary, who, after going to India with the 6th Devon Regiment in 1914, was killed in action in Mesopotamia in March last. Captain Dunn Pattison was the first officer connected with the institution to lose his life during the war, and a bronze tablet is to be fixed in the vestibule of the infirmary to commemorate his services. The dispenser, Mr. Blackmore, has been acting as honorary secretary, and the post of house surgeon has been filled by a Belgian medical officer, Dr. Vermeylen. The war has thus left a mark on the staff of the institution which mere departures for active service, serious though they are, would not cause.

### THE TREATMENT OF MEASLES IN LONDON.

Considerable dissatisfaction has been expressed by several Metropolitan Boards of Guardians at the attitude adopted by the Metropolitan Asylums Board in allocating a much smaller number of beds for cases of measles. The policy adopted by the managers in 1910 was to utilise accommodation as it became available, owing to the reduction in the number of patients under treatment, for the reception of cases of measles and of whooping-cough. This accommodation is primarily reserved for poor children, as it is considered that they have the first claim on the managers' resources, and only when there is more accommodation available than is required for Poor-Law patients are other cases to be received. Since the outbreak of war many cases of measles and German measles occurring amongst the troops have been received on the application of the Army authorities, though fortunately the requirements of the latter have not come up to expectation, so that at the present time, owing to this and the gradual drop in scarlet fever, there are certain wards not being used in most of the hospitals. The time of the year has now been reached when an increase may soon be expected in the number of scarlet-fever admissions, and therefore the managers have come to the conclusion that it would be inadvisable to allocate more accommodation for measles cases even if the desire were more general than it appears to be. Another factor to be contended with is that it has become increasingly difficult to secure staff of all grades under existing conditions.

### THE VALUE OF A LADIES' AUXILIARY COMMITTEE.

The splendid services which may be rendered to a hospital by an energetic ladies' auxiliary committee is exemplified in the happy financial position in which the Manchester Children's Hospital at Pendlebury finds itself. The hospital's area is mapped into districts by the ladies' auxiliary, and these districts are so admirably worked that twenty-three of them each provided £50 for the maintenance of a cot last year, six maintained two cots, and one supported three cots. In all, the hospital received nearly £2,000 through the efforts of its ladies' auxiliary. Taddington Hall, near Miller's Dale, in Derbyshire, which has been given by Mr. and Mrs. Bramwell for a convalescent home, has now been legally conveyed to the hospital's trustees. The hall stands in grounds extending to about six acres.

### THE R.A.M.C. DEPOT MAGAZINE.

This magazine, the first number of which we noticed some two or three months ago, has quite evidently "made good." It has issued twelve numbers; and, if we may be allowed to say so, there has been a distinct improvement in quality throughout the series. The Editor is of the same opinion, which he does not hesitate to avow; it is pleasing to find that his efforts are widely appreciated among the rank and file of the Corps, as evidenced by a healthy financial position and an expanding circulation. The magazine is especially rich this week in photographs, though the cartoonists have not allowed themselves to be entirely superseded by the camera. Personalities are a smaller feature than they were in earlier numbers, which is all to the good, though probably most of the subscribers would be sorry to see them dropped altogether. The *D.M.*, as the Editor loves to call his paper, is certainly a good pennyworth.

### THE CONTENTS OF YOUR HOSPITAL GAZETTE.

For the information of hospitals with a lower standard of editing we take the opportunity afforded by the opening of a fourth volume to enumerate the principal contents of the August number of the *Craigleith Hospital Chronicle*. First let us take the delightful paper by the Rev. Thomas Hannan, C.F., in which he describes from personal knowledge the country "where our soldiers are in Switzerland," a subject of topical interest so agreeably treated as to awaken pleasant memories in many, and hopes of future visits to us all. Then a patient, seemingly a medical man, gives his impression of nurses as the result of several illnesses, under the question-begging title of "Angels." Another writer describes the "Germans on Holiday" whom he met before the war and Dr. J. A. MacDougall concludes his series on the "Scottish Regiments" with an article on "The Seaforth Highlanders." A curious account is also given of "Some Unusual Occupations Followed by the Blind," which will be read eagerly and, let us add, hopefully, by many. The occupations include those of a coal agent, a clergyman, flower-sellers, and typists. Then there are the usual features of verse, illustration, hospital notes, plan of the new recreation hut, and so on. It is an impressive list, and for those editors who can think of no subjects that might not appear in *Comic Cuts* we are happy to add that the *Chronicle* has produced a surplus profit of £70 on the previous two volumes, after having paid £2 each per month to the library and tobacco funds.

### A DUBLIN CHARITY AND THE LATE REBELLION.

One of the Dublin charities which performs, among other things, Samaritan work in connection with the Dublin hospitals is the Mendicity Institution. This, as its disagreeable name implies, is an old-established organisation which sends patients back to their homes when they are unable to find their fares on being discharged, and also helps those who for a similar reason are unable to return home from the metropolis after a vain search for work, or can only find it at a distance. The institution had a lively time during the Sinn Fein Rebellion, for it was occupied by Sinn Feiners and eventually considerably injured. As a result of the wrecking a claim for full compensation has been lodged, to repay for the rebuilding of the old institution.

### AN ASYLUM FARM.

An interesting report dealing with food problems at the County Asylum, Radcliffe-on-Trent, was presented last week to the Nottingham County Council. References were made to changes in the dietary, both for the patients and the staff, which at the time they were made were not regarded at all favourably by those concerned. The alterations were accepted as a condition due to the war, however, but it is reported that the substitutes adopted in lieu of butchers' meat twice a week are now really appreciated. In consequence, it is proposed to continue the new dietary after the war. All the milk, vegetables, and fruit, and also small quantities of the meat required by the asylum, are supplied from the institution's own farms, which, in addition, sold produce during 1915 to the value of £2,234 to outside consumers. Last year the farms made the handsome profit of £1,508.

### AN IRISH MEMORIAL TO EDITH CAVELL.

The Board of the Royal City of Dublin Hospital have received a cheque for £200 from the honorary secretary of the Edith Cavell Memorial, for the purpose of ultimately endowing an Edith Cavell bed in the institution. A bed therefore is being opened and named without delay, though it is still hoped that the further sum of £300 may be raised so as to make the endowment a permanent one. It is an opportunity for the women of Ireland which will soon pass if it be not acted upon, for in a great war like the present events follow one another in quick succession. While these lines are being written, in fact, Captain Charles Fryatt's fate is announced, and already comparisons are being made between it and Edith Cavell's.

### MANOR HOSPITAL AS A WAR HOSPITAL.

Representations having been made by the Board of Control, on behalf of the Army Council, as to the urgent need for the provision of additional hospital accommodation for wounded and sick soldiers, the London Mental Hospitals Committee have agreed to the Manor Mental Hospital at Epsom being emptied of lunatic patients and prepared for use as a war hospital. The patients are being removed to other county or London mental hospitals, and some two hundred suitable cases will be sent to the institutions of the Metropolitan Asylums Board. As in the case of the Horton Mental Hospital, which is at present being used as a war hospital, the Army Council has undertaken to meet all charges in connection with the adaptation of the building to its new use, the maintenance and repair of the premises, the reinstatement of the premises at the end of occupation, and the additional equipment found to be necessary. The Army Council will also refund all expenses in connection with the administration of the hospital and all expenses incurred in connection with the transfer and accommodation in the other institutions of the patients who were at the Manor Mental Hospital.

### MORE WOMEN WORKERS AND NURSES WANTED.

The Hon. Arthur Stanley and Lord Ranfurly, on behalf respectively of the British Red Cross Society and the Order of St. John, in the " Letter Box " state that an urgent necessity has arisen for more V.A.D. nursing members (women), and V.A.D. general service members, in military and auxiliary hospitals at home. The demands of the military authorities for nurses are very heavy, and cannot be met out of the existing supply. Nor is the demand likely to diminish, for a long time at least. All women who are not giving the whole of their time and service to the war, and have no ties to prevent them, are appealed to for help in this emergency; and it is impossible to believe that this appeal will fall upon deaf ears. Unquestionably there are many hundreds of women by whom this call can be answered. Let the facts be brought sufficiently home to them and the necessary workers will offer their services.

### A LEGACY TO A BOARD OF GUARDIANS.

It is often said that when an institution is supported by public funds, voluntary interest and generosity are suspended, and the truth is general enough to make exceptions interesting apart from the fact that machinery can be invented, as in the Colonial hospital system, to combine the two. Even Boards of Guardians receive legacies on occasion. For example, the Charity Commissioners have informed the Hastings Guardians that the late Mr. W. Gilbert, a resident, has left £50 on trust, the income from which is to be applied to the annual pleasure day and Christmas treats for the inmates. It is true that the solicitors have announced that the testator's intentions can only be fulfilled to the extent of £31, but the intention was there, and the chairman of the board hopes that other residents will follow Mr. Gilbert's example. The chief value of the gift lies in reminding this and other boards that the public is interested in maintaining as high a standard as possible, and in thereby encouraging the further humanisation of the Poor Law.

### THE COCAINE PROCLAMATION

A Royal Proclamation has been issued prohibiting the importation of cocaine and of opium, except under licence from the Government; and an Order in Council has been issued containing drastic regulations which should suffice to stamp out the traffic in these drugs amongst soldiers and others. We deal with the position fully on another page. These regulations relate more particularly to the distribution of cocaine, and make it an offence to be in possession of this drug except under certain stated conditions. The gift of cocaine, equally with the sale of it, becomes an offence; this important proviso should go far to help in suppressing the cocaine traffic, for hitherto the proving of a sale was often difficult or impossible. The regulations also guard against the abuse of physicians' prescriptions by clauses requiring a fresh signature by a medical man before the prescription can be dispensed afresh: this plan has long been advocated in The Hospital, and it is gratifying to see it put in force at last. The spread of the cocaine habit seems to be attributable to the large influx of Canadian soldiers, who, in turn, acquired it from the United States. Some authorities, we note, ascribe the growth of the habit to the restrictions imposed on alcoholic refreshment. Certainly if human nature must indulge either in drink or drugs, it is better that it should be the former; for moderate drinking is the rule, not the exception, whereas moderation to the drug slave is unknown.

### DECORATIONS FOR DOCTORS FROM WITTENBERG CAMP.

At a Chapter-General of the Order of St. John held at St. John's Gate last week, Lord Plymouth, the Sub-Prior, presented gold life-saving medals of the Order to Major Harold Edgar Priestley, C.M.G., R.A.M.C., and Captain Alan Cunliffe Vidal, D.S.O., R.A.M.C., in recognition of the gallantry displayed by them at the Wittenberg Camp. Captain James La Fayette Lauder, D.S.O., R.A.M.C., was prevented by absence abroad on military duty from attending to receive a similar medal. Lord Plymouth also presented a silver medal to Mrs. Selby for conspicuous bravery.

### THIS WEEK'S DRUG MARKET.

The market has been quiet, and with the exception of the buying of drugs for the Medical Service of the Army, which is always going on, there has really been very little doing. Buyers are rather nervous after the heavy drop in bromides, and in face of the downward tendency in the prices of various other important drugs; they are only buying when the depletion of their stocks renders this necessary. There has been a further fall in bromides, and it has been possible to buy even at something a little below makers' reduced prices. Salicylates continued to tend downwards in price by slow degrees. Acetylsalicylic acid is distinctly lower, and any falling off in the demand would probably be accompanied by a further reduction in price. Acetanilide is also again lower in value. Benzoic acid is difficult to obtain, and holders of sodium benzoate ask very high prices. It remains to be seen what effect the cocaine regulations will have on the price of cocaine. Tartaric acid is somewhat cheaper, but there is little change in the position of citric acid. Phenacetin is still difficult to obtain, and very high prices continue to be asked. At the recent public sale of drugs in Mincing Lane there was a poor demand for the rather large supplies offered; good prices were realised for camphor, honey was cheaper, and senna leaves were slightly lower.

